# Forms and Lability of Phosphorus in Algae and Aquatic Macrophytes Characterized by Solution ^31^P NMR Coupled with Enzymatic Hydrolysis

**DOI:** 10.1038/srep37164

**Published:** 2016-11-16

**Authors:** Weiying Feng, Yuanrong Zhu, Fengchang Wu, Zhongqi He, Chen Zhang, John P. Giesy

**Affiliations:** 1 State Key Laboratory of Environmental Criteria and Risk Assessment, Chinese Research Academy of Environmental Sciences, Beijing 100012, China; 2 College of Water Sciences, Beijing Normal University, Beijing 100875, China; 3 USDA-ARS, Southern Regional Research Center, New Orleans, LA 70124, USA; 4 Department of Biomedical Veterinary Sciences and Toxicology Centre, University of Saskatchewan, Saskatoon, SK S7N 5B3, Canada

## Abstract

Solution Phosphorus-31 nuclear magnetic resonance (^31^P NMR) spectroscopy coupled with enzymatic hydrolysis (EH) with commercially available phosphatases was used to characterize phosphorus (P) compounds in extracts of the dominant aquatic macrophytes and algae in a eutrophic lake. Total extractable organic P (P_o_) concentrations ranged from 504 to 1643 mg kg^−1^ and 2318 to 8395 mg kg^−1^ for aquatic macrophytes and algae, respectively. Using ^31^P NMR spectroscopy, 11 P_o_ species were detected in the mono- and diester region. Additionally, orthophosphate, pyrophosphate and phosphonates were also detected. Using EH, phytate-like P was identified as the prevalent class of enzyme-labile P_o_, followed by labile monoester- and diester-P. Comparison of the NMR and EH data indicated that the distribution pattern of major P forms in the samples determined by the two methods was similar (*r* = 0.712, *p* < 0.05). Additional ^31^P NMR spectroscopic analysis of extracts following EH showed significant decreases in the monoester and pyrophosphate regions, with a corresponding increase in the orthophosphate signal, as compared to unhydrolyzed extracts. Based on these quantity and hydrolysis data, we proposed that recycling of P_o_ in vegetative biomass residues is an important mechanism for long-term self-regulation of available P for algal blooming in eutrophic lakes.

Lake eutrophication is a serious environmental concern in world, including the lakes from middle and lower reaches of the Yangtze River watershed and southwest China^1^,^2^,^3^. Limiting phosphorus (P) supply to water can be an effective strategy for restoration of eutrophic lakes^1^. While the reduction of P inputs from industrial and agricultural sources is required for this purpose, internal recycling of death plants-associated P and sediment-associated P can slow eutrophic lake remediation^4^,^5^,^6^. Previous studies on the role of internal P cycling in lake eutrophication and its remediation have focused primarily on soluble and sediment-associated inorganic P (P_i_)^7^,^8^,^9^,^10^. However, organic P (P_o_) is also an important component of total phosphorus (TP) in the overlying water^11^, sediments^12^ and debris derived from decomposition of algae and aquatic plants^13^. The P_o_ concentration in biomass from algae and aquatic macrophytes was found to be 12.9-fold and 1.8-fold greater than in sediments, respectively^13^, which represents a significant source of internal P for a eutrophic lake. In aquatic systems, P_o_ can be converted to bioavailable P (e.g., 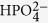) for algal blooming through a series of redox-driven solubilization reactions and phosphatase-mediated hydrolytic processes^14^,^15^. In addition, some forms of P_o_ can be directly assimilated by algae^16^. Thus, P_o_ plays a key role in nutrient availability and algal blooming in lakes, irrespective of attempts to limit external P inputs. Despite the importance of lake internal P_o_ in the eutrophication process, the P composition of algae and aquatic macrophytes and their contribution to internal loading and cycling of P in lakes remains poorly understood.

In the last few decades, phosphorus-31 nuclear magnetic resonance spectroscopy (^31^P NMR) has proven to be a useful tool to identify and quantify P_o_ in samples from environmental samples^3^,^17^,^18^,^19^,^20^,^21^. Three major classes of P_o_ have been identified in aquatic environments: orthophosphate monoesters, orthophosphate diesters and phosphonate^9^,^13^,^22^,^23^. Characterization of P_o_ with ^31^P NMR can identify specific orthophosphate esters, such as sugar phosphates, inositol phosphates [IHP], phospholipids, and nucleic acid [deoxyribonucleic acid (DNA) and ribonucleic acid (RNA)]^21^,^24^. Enzymatic hydrolysis (EH) with commercially available phosphatases has also been used to characterize P forms and evaluate their bioavailabilities in environmental samples, including animal manures, soils and sediments, and water^15^,^17^,^21^,^25^,^26^,^27^. For example, bioavailable P_o_ forms, such as monoester P, diester P and phytate-like P, were functionally classified using combinations of three commercially available phosphatases: alkaline phosphatase (AP), phosphodiesterase (PDE), and phytase (Phy)^2^. Importantly, the combination of solution ^31^P NMR spectroscopy and EH has been applied to provide supplemental and integrated information on P forms and their labilities in environmental samples^9^,^16^,^17^,^28^.

In the current study, solution ^31^P NMR spectroscopy coupled with EH was applied to analyze P forms and their bioavailabilities in the debris derived from algae and aquatic macrophytes in lakes. The objectives of the study were to i) quantitatively compare differences in P forms (extracted by NaOH-EDTA) of common species of algae and aquatic macrophytes by solution ^31^P NMR and EH, and ii) evaluate potential biogeochemical cycling of P_o_ derived from algae and aquatic macrophytes in Lake Tai, a eutrophic lake in the lower reaches of Yangtze River watershed, China. The eventual goal of this study is to better understand internal P loading and cycling relevant to lake eutrophication and remediation.

## Results and Discussion

### Forms of phosphorus in aquatic macrophytes and algae characterized by solution ^31^P NMR

With 0.5 M NaOH + 25 mM EDTA as the extracting agent, the recovery of TP and P_o_ in aquatic macrophytes was 92.4% and 88.1%, respectively. The recoveries of TP and P_o_ from algae were 96.4% and 90.8%, respectively. The application of the NaOH-EDTA extraction technique on algae and aquatic macrophytes for solution ^31^P NMR analysis was reported in a previous work^13^. The recoveries of TP and P_o_ in algae and aquatic macrophytes in the current and previous study were similar, which indicated sample homogeneity and repeatability of the extraction procedure.

**Figure 1:**
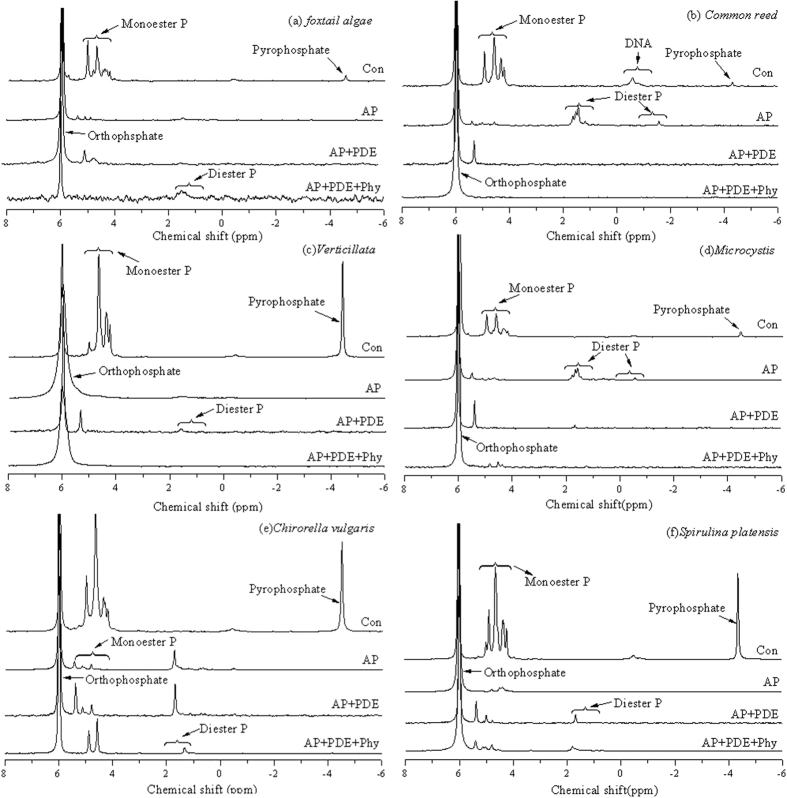
Solution ^31^P NMR analysis of the NaOH-EDTA extracts of aquatic macrophytes and algae before (Con) and after treatments with AP, AP + PDE and AP + PDE + Phy enzymes.

Solution ^31^P NMR spectra of NaOH-EDTA extracts of aquatic macrophytes and algae are shown in Fig. 1 (i. e., Con: Control without EH). Integration of these peaks, and chemical shifts in the spectra, provided quantitative data of specific P species in the six aquatic organisms samples (Supplementary Information, S.I. Table 1). Up to 11 different P_o_ species were identified (Table 1, Fig. 1). Concentrations of extracted P_o_ ranged from 504 to 1643 mg kg^−1^ in aquatic macrophytes and from 2318 to 8395 mg kg^−1^ in algae. These data indicated that there was a large difference in P content between aquatic macrophytes and algae, but the relative distribution (i.e., percentage) of specific forms of NaOH-EDTA extractable P_o_ was similar among the two sample types. The detected P_o_ forms included monoester P, diester P and phosphonate. Monoester P (the sum of phytate and other monoester P) comprised the largest P_o_ fraction. The percentage of phytate in NaOH-EDTA extractable P in non-hydrolyzed samples ranged from 5.6 to 41.9% (average of 23.0%). The percentage of other monoester P in these samples ranged from 8.6 to 44.3% (average of 22.6%). Other monoester P was present as glycerides (i.e., α- and β-glycerophosphate), nucleotides (i.e., guanosine 2′ monophosphate, cytidine 5′ monophosphate, adenosine 5′ monophosphate) and sugars (i.e., fractose 6-phosphate, glucose 1-phosphate and glucose 6-phosphate) (Table 1, Fig. 1). Among them, glycerophosphate was assumed to derive from spontaneous hydrolysis of phospholipids in the alkaline extracts, as phospholipids constituted the main component of plant cell membranes^13^,^29^. The nucleotide category includes the degradation products of RNA and possibly DNA, both of which are involved in cellular metabolic and reproductive activities^19^. Sugar phosphates are important components in providing energy for cellular processes. This wide variety of monoester P forms found in macrophytes and algae has also been found in lake sediments and water^2^, implying that debris from decomposed algae and macrophytes were released into the lake water body and deposited as sediments. The percentage of diester P in extracts was generally low compared to monoester P (Table 1, Fig. 1). A small amount of phosphonate was detected in *Spirulina*. Orthophosphate and pyrophosphate were the forms of P_i_ detected by NMR analysis and found in all samples investigated. The percentage of orthophosphate and pyrophosphate in non-hydrolzyed samples ranged from 27.1% to 67.5% and from 0.9 to 18.8%, respectively.

In order to compare NMR data with the P identities revealed by EH, we generalized the NMR-identified P species into six categories (i.e., orthophosphate, phytate, other monoester P, diester P, pyrophosphate and phosphonate). In aquatic macrophytes, the majority of extracted P was orthophosphate (27.3 to 56.6% of total extracted P), phytate (12.2 to 38.9%), other monoester P (10.8 to 33.5%), diester P (1.6 to 6.2%) and pyrophosphate (0.9 to 18.8%). Similarly, algae were dominated by orthophosphate (27.1 to 67.5%), phytate (5.6 to 41.9%), other monoester P (8.6 to 44.3%), diester P (1.3 to 14.4%), pyrophosphate (1.8 to 2.3%), with minor amounts of phosphonate (0 to 0.7%).

### Forms of phosphorus in aquatic macrophytes and algae characterized by enzymatic hydrolysis

Using EH, P in NaOH-EDTA extracts was classified into five groups: P_i_, labile monoester P, diester P, phytate-like P, and enzyme-stable P (Table 2). Although the content of extractable P_i_ was greater in algae (3220 mg kg^−1^) than in macrophytes (1126 mg kg^−1^), the percentage of total extractable P present as P_i_ was lower in the algae sample (35.5%). For P_o_, aquatic macrophytes contained an average of 180 mg kg^−1^ labile monoester P, which accounted for approximately 8.0% of extracted P. Labile monoester P was present at an average of 1566 mg kg^−1^ in algae samples, which accounted for approximately 16.5% of extracted P. *Spirulina* (an algal species) contained diester P at 695 mg kg^−1^ (~6.8% of extracted P). *Common reed*, a macrophyte, contained 45 mg kg^−1^ diester P (~3.8% of extracted P). The content of phytate-like P of macrophytes was 380 mg kg^−1^ (~14.0% of extracted P) in macrophytes, and 1235 mg kg^−1^ in algae (~14.5% of extracted P). Concentrations of enzyme-stable P in aquatic macrophyte and algal extracts accounted for 25.7% and 31.2% of extracted P, respectively.

The general distribution pattern of P was P_i_ > enzyme-stable P > phytate-like P > labile monoester P > diester P in NaOH-EDTA extracts of both aquatic macrophytes and algae. The distribution pattern was consistent with NaOH-EDTA extractable P from soil^30^, dairy manure^17^, and lake sediments^2^, but different from that of poultry manure^17^. In general, phytate-like P represented the largest proportion of enzyme-hydrolysable P, followed by labile monoester P and diester P. Contents of total hydrolysable P_o_ ranged from 140 to 951 mg kg^−1^ in aquatic macrophytes and 1369 to 4387 mg kg^−1^ in algae. Additionally, 238 to 975 mg kg^−1^ and 2324 to 3726 mg kg^−1^ of enzyme-stable P, respectively, in macrophytes and algae could not be hydrolyzed by the enzymes used in this study. The percentage of enzyme-stable P in extracted P of aquatic macrophytes were similar to that of animal manure^26^ and lake sediments^9^.

The percentage of enzyme-stable P in extracted P of algae was slightly higher than macrophytes, perhaps due to differing abilities of the two organism types to mineralize P_o_^8^. Enzyme-stable P in NaOH-EDTA extracts likely consisted of P_o_ in high molecular weight (e.g., humic acid) or combined with high molecular weight with metal bridge, which would be inaccessible by active sites of enzymes^3^,^30^. The percentage of P_i_ in extracted P of macrophytes was slightly lower than that reported for lake sediments (60.9%)^9^ and were consistent with previous reports^31^. The percentages of P_i_ in extracted P of algae were lower than aquatic macrophytes likely due to the more complex cellular and organ systems of macrophytes. Thus, more P_i_ would exist in complex forms than in algae.

### Comparison of P forms by ^31^P NMR and Enzymatic Hydrolysis

**Figure 2:**
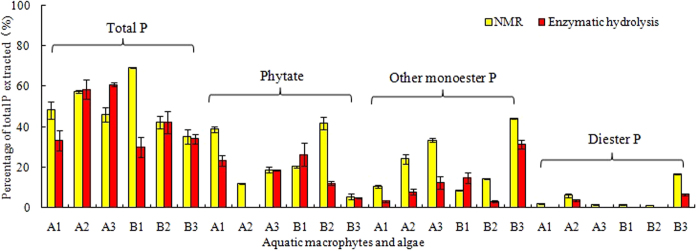
Comparison of P forms in the NaOH-EDTA extracts of aquatic macrophytes and algae biomass samples identified by solution ^31^P NMR spectroscopy and enzymatic hydrolysis. A1, A2, A3, B1, B2, and B3 stand for *Foxtail algae, Common reed, Verticillata, Microcystis, Chiorella vulgaris and Spirulina*, respectively.

Three aquatic macrophytes were *Foxtail algae* (A1), *Common reed* (A2), *Verticillata* (A3), respectively, and three algal species were *Microcystis* (B1), *Chirorella vulgaris* (B2), *Spirulina* (B3), respectively. Four forms of P in aquatic macrophytes and algae were similarly identified by ^31^P NMR and EH: (1) orthophosphate or P_i_, (2) phytic acid (phytate) or phytate-like P, (3) other monoester P or labile monoester P, and (4) diester P. There was no significant difference in P_i_ content among three samples (A2, B2, B3) measured by the two methods, but there was a significant difference in P_i_ content in other three samples (A1, A3, B1) measured by the two methods (Fig. 2). On the whole, the quantity of P_i_ measured in the aquatic macrophytes and algae were slightly higher with ^31^P NMR (~49.8% of total extracted P) than EH (~43.3%). The content of phytate detected by the two methods was similar for three out of the six samples. Phytate content was lower in the NaOH-EDTA extracts of A1 and A2 and B2 with EH than measured with NMR. Using EH, significantly other monoester P was detected in the extracts of both the macrophytes and algae than with NMR. Similarly, the percentage of diester P determined by EH was less than by ^31^P NMR, with the exception of B1. This may be attributed to the presence of unstable diester P that was easily biologically degraded to monoester P^32^. Other studies have shown the rapid hydrolysis (16 h) of RNA or bacterial compounds in alkaline extracts^19^,^32^ and plant tissues^33^. In a word, not all P forms (i.e., enzyme-stable P) are easily hydrolyzed by commercially available phosphatases, they may be lack of specificity and non-ideal incubation conditions. Approximately 25.7% and 31.2% of total extractable P in macrophytes and algae were characterized as enzyme-stable P in aquatic macrophyte and algae, respectively (Table 2). As a result, these causes led to the differences in total detectable P_o_ concentrations between the NMR and EH methods.

Overall, these data indicated that the distribution patterns of major P forms in aquatic macrophytes and algae determined by EH and solution ^31^P NMR were similar (*r* = 0.712, *p* < 0.05, n = 5). While NMR provided information on the relative abundance of specific P compounds and classes, it is not an economically viable approach for many researchers and requires expert interpretation of the resulting spectra^9^. Enzymatic hydrolysis provided a valid estimate of hydrolyzable, and thus potentially bioavailable 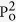. However, the EH method also has some disadvantages. For example, the EH method may not effectively hydrolyze some forms of P_o_ (i.e., enzyme-stable P). Besides, P was only classified into five groups in the EH method (Table 2). In contrast, NMR analysis could identify 18 specific P compounds (Table 1). In this study, the combination of EH and NMR for analyses of specific P forms and bioavailability in aquatic macrophytes and algae were both complimentary and supplementary and provided detailed information related to endogenous P cycling in eutrophic lakes.

### Lability of phosphorus in aquatic macrophytes and algae characterized by ^31^P NMR coupled with enzymatic hydrolysis

**Figure 3:**
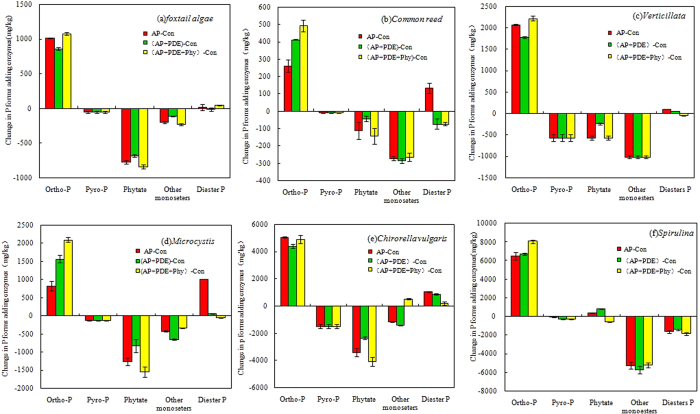
Degradation characteristics of P in aquatic macrophytes and algae revealed per ^31^P NMR spectroscopic changes of the NaOH-EDTA extracts due to the EH treatments. These treatments were added AP, AP + PDE and AP + PDE + Phy enzymes.

Incubation of NaOH-EDTA extracts of macrophytes and algae with commercially available phosphatases clearly changed peak distribution in the NMR spectra (Fig. 1). Addition of any phosphatase treatment (i.e., AP, AP + PDE, or AP + PDE + Phy) resulted in decreased NMR signal in the monoester (i.e., phytate and other monoester P) and pyrophosphate regions, with a corresponding increase in orthophosphate (Figs 1 and 3). As an example, the NMR spectral features of *foxtail algae* (A1) with three types of enzyme treatments were analyzed in detail (Table 1). Addition of AP significantly increased measured orthophosphate by 1014 mg kg^−1^ (46.9%) compared to Control and significantly decreased concentrations of some P_o_ forms by 44.5% (phytate-35.9%; other monoester P-9.3%) and pyrophosphate by 2.4% in *foxtail algae*. There was a small increase in diester P (0.7%) after hydrolysis by AP (Table 1, Fig. 3). On the other hand, the peaks assigned to chiro-IHP, neo-IHP, Guanosine 2′ monophosphate, AMP and DNA did not appear after the AP enzyme treatment. In addition, a new peak appeared at a chemical shift of 4.58 ppm in the monoester region after the treatment, which was assigned to O-phosphorylethanolamine (P-ine). Pyrophosphate, comprising 2.4% of total P in the untreated extract (the peak at −4 to −5 ppm), was completely hydrolyzed by the AP treatment (Table 1, Fig. 3).

Likewise, treatment with AP + PDE resulted in the complete loss of pyrophosphate along with some signals in the monoester region (3.5 to 6.0 ppm). Adding AP + PDE significantly decreased P_o_ forms by 39.8% (phytate-31.5%; other monoester P-8.9%; diester P-0.7%) and significantly increased orthophosphate by 861 mg kg^−1^ (Table 1, Fig. 3). Treatment with AP + PDE additionally removed diester P (−1 to 2 ppm chemical shift), while treatment with AP alone did not affect this peak. Solution ^31^P NMR analysis of the enzyme-treated extracts of *foxtail algae* (Fig. 1) showed that the anticipated substrate specificity of AP + PDE was also achieved in foxtail algae extracts. Specifically, in the extracts of *foxtail algae*, hydrolysis caused a significant increase in orthophosphate by 1078 mg kg^−1^ and a small increase in diester P by 44 mg kg^−1^ (2.1%) with a significant decrease in other three P forms by 52.1% (phytate-38.9%, other monoester P-10.8%, and pyrophosphate-2.4%).

Adding AP alone and AP + PDE + Phy slightly increased detected diester P (Fig. 3). This observation might be due to the fact that glycerophosphate in extracts was derived from phospholipid hydrolysis during alkaline extraction^29^. In the current case, this process was likely to be reversed by phosphorylation activity^2^,^21^. Interestingly, guanosine 2′ monophosphate and α-glycerophosphate were completely hydrolysed by the AP and AP + PDE + Phy (Table 1). A portion of these compounds may have been transformed to diester P, which is supported by the measured increases in diester P with AP alone and AP + PDE + Phy treatments. The three P_o_ classes were quantified by the increases in *molybdate-reactive P (i. e. MRP)* in enzyme treated samples compared to the control (non-enzyme amended) (all values in mg kg^−1^ of *foxtail algae* dry biomass) as phytate: 841, other monoester P: 233, diester P: 44. In addition, the NMR spectra showed some new peaks with enzyme treatments, as shown in Table 1, such as glucose 6-phosphate (B1 + 1e), cytidine 5′ monophosphate (B1 + 1e, + 3e, B3 + 3e), AMP (A2 + 3e), 3-sn phosphatidic acid (P-acid) (A1 + 1e, etc.) and P-ine (B1 + 1e, B2 + 1e, + 3e). Phytate has many stereo-isomers, of which chiro-, neo-, myo-, scyllo-IHP have been found to be most common in soils^21^ and sedments^5^. In this study, chiro-IHP and neo-IHP were identified in macrophytes and algae (Fig. 1, Table 1). While only a portion of the exacted phytate with EH during the 16 h incubation in the current study, in this work, most of the phytate disappearance was reported after a 40 d incubation under anaerobic conditions^34^.

**Figure 4:**
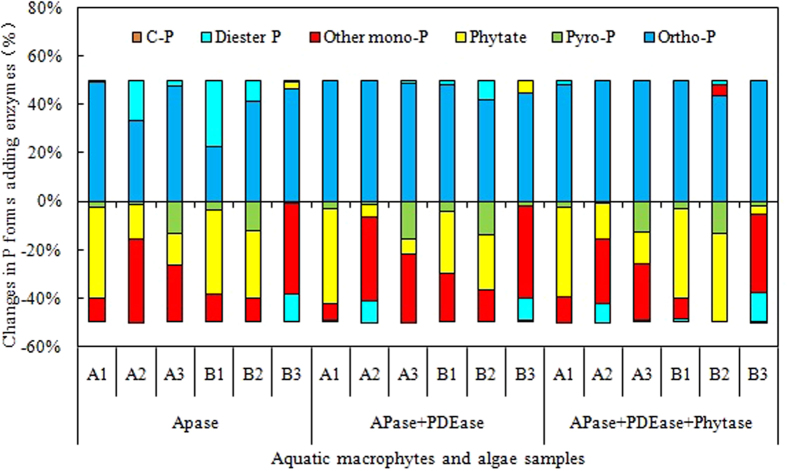
Percentage changes of P forms, as proportion of total extractable P, in aquatic macrophytes and algae after the treatments with AP, AP + PDE and AP + PDE + Phy enzymes.

Overall, the vast majority of monoester P, including phytate, and total pyrophosphate were transformed into orthophosphate with EH. As shown in Fig. 4, Phytate (average of 18.9%, of extractable P), other monoester P (20.7%) and pyrophosphate (6.6%) were transformed into orthophosphate with AP. Phytate (12.3%), other monoester P (20.8%) and pyrophosphate (7.0%) were transformed into orthophosphate with AP + PDE. Phytate (22.8%), other monoester P (17.7%) and pyrophosphate (7.0%) were transformed into orthophosphate with AP + PDE + Phy. These data suggests high potential for release of monoester P and pyrophosphate during decomposition of macrophytes and algae and that these P forms could be further enzymatically converted into orthophosphate in aquatic systems. In most instances, P is a limiting nutrient in freshwater systems and orthophosphate concentrations are linked to cyanobacterial proliferation in eutrophic lakes^35^. In contrast, some forms of P_o_ are bioavailable to algae. Therefore, this cyclic process would be cause algae outbreak again. These results suggest that bioavailable P_o_ derived from decomposing aquatic macrophytes and algae could play an important role in maintaining the eutrophic status of lakes and could present complications to mitigation efforts.

### Algal blooming and biogeochemical cycling of phosphorus in lakes

**Figure 5:**
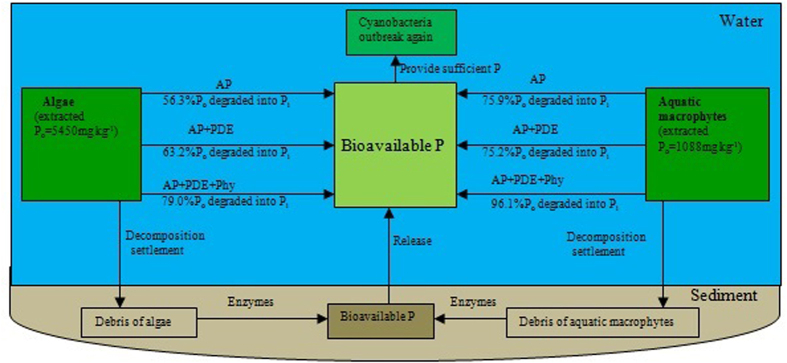
A schematic diagram of the cycling process of organic phosphorus (P_o_) of aquatic macrophytes and algae in freshwater lakes. The proportions were percentage of hydrolyzed P_o_ in NaOH-EDTA extractable P_o_.

Based on results in this study, biogeochemical cycling of P driven by algal blooming in eutrophic Lake Tai was further discussed (Fig. 5). Approximately 32.7% to 41.3% and 39.5% to 46.0% of extractable P_o_ from algae and macrophytes, respectively, has potential for phosphatase hydrolysis to soluble orthophosphate and released into the water bodys. Orthophosphate can be directly assimilated and utilized by cyanobacteria and other organisms in eutrophic lakes. The majority of P_o_ from decomposition would be deposited as sediments, which can then be or transformed into orthophosphate under the action of various enzymes and microorganisms within sediments and subsequently released to overlying water. Both water and sediment sources of bioavailable P could provide sufficient P for proliferation of algae and cyanobacteria. In turn, P incorporated into biomass during algal blooms could be readily replenished by the bioavailable P from the debris of aquatic macrophytes and algae. Thus, P cycling among sediment, water, and aquatic organisms is the primary mechanism of continuous (or repeated) algal and maintenance of eutrophic conditions.

As the biomass of algae and aquatic macrophytes were 13.57 mg kg^−1^and 2606 g m^−2^ in Lake Tai in 2012, which has a water volume of 44.3 × 10^8^ m^3^ and area of 2338 km^26^. From this information, we estimated the total biomass of algae and aquatic macrophytes in Lake Tai were 6.01 × 10^4^ Mg and 6.09 × 10^6^ Mg, respectively. With the P_o_ levels of algae and aquatic macrophytes we measured in this study (Fig. 5), we calculated the P_o_ biomass of algae and aquatic macrophytes to be approximately 328 Mg and 7510 Mg, respectively. Of this amount of P_o_, algae and macrophytes would contain 107 to 135 Mg and 2966 to 3455 Mg, respectively, of bioavailable P. This bioavailable P would be released into the water body and promote repeated algal blooms. Therefore, the contribution of P in the debris from aquatic organisms must be considered in relation to a endogenous P cycling in eutrophic lakes.

It is reported that cyanobacteria salvage (a technology for removal of cyanobacteria from the eutrophic lakes by a salvage ship or machine) could effectively eliminate the phenomenon of algal blooming so that cyanobacteria salvage could reduce the concentration of nitrogen (N) and P in lakes^36^. In addition to the physical methods, cyanobacteria (e.g., *Microcystis aeruginosa*) could be removed efficiently by using quaternary ammonium salt of Gemini surfactant^37^. In order to prevent the release of P from the debris of aquatic macrophytes, which continue to proliferate and cause secondary pollution in lakes, the debris of aquatic macrophytes must be harvested in a timely fashion. Harvested aquatic organisms could be mechanically broken down and recycled as a source of organic fertilizer for crop production^38^. No matter what measures to take, the importance on preventing or reducing the P release from the debris of algae and aquatic macrophytes would make great contribution on controlling the phenomenon of eutrophication of lakes and repeated algal blooming from internal P cycling.

## Conclusions

About 90% of TP and P_o_ in debris of algae and aquatic plants could be extracted by 0.5 M NaOH-25 mM EDTA. Solution ^31^P NMR analysis found 11 P_o_ species in the mono- and diester region plus orthophosphate, pyrophosphate and phosphonate in these extracts. The bioavailability of these NaOH-EDTA extractable P components was further characterized by EH, which revealed that 2173 mg kg^−1^, accounting for 28.3% of extracted P, was hydrolyzable with commercially available phosphatases. The hydrolyzable portions included phytate-like P (1809 mg kg^−1^, 14.2% of extracted P), labile monoester P (808 mg kg^−1^, 12.3% of extracted P) and diester P (123 mg kg^−1^, 4.9% of extracted P). However, 1657 mg kg^−1^, (~28.5%) of extracted P, was not enzyme hydrolyzable (enzymatic stable P).

Based on the results of ^31^P NMR spectroscopic analysis of EH extracts, most proportions of P_o_ and pyrophosphate were hydrolyzable by phosphatases into orthophosphate. Enzymatically stable P was assumed to be macromolecular P_o_ that would accumulate and be preserved in lake sediments for long periods. According to lability of these P_o_ forms, recycling of P_o_ in the debris derived from aquatic macrophytes and algae, especially algae, would be an important internal source of bioavailable P in lakes. Recycling of concentrated bioavailable P in aquatic macrophytes and algae would continuously support algal blooming in a eutrophic lake, such as Lake Tai.

## Materials and Methods

### Study Site and Sample Preparation

Lake Tai (33°55′−31°32′N, 119°52′-120°36′E), a large shallow, eutrophic lake, is located in Jiangsu Province, China. Lake Tai was chosen as a study site because it is one of the eutrophic lakes where the algal blooms have been closely monitored for decades^39^. Research indicated that the water column in the algal blooming areas contains a high level of aquatic macrophytes and phytoplankton^31^,^38^. In an earlier study^13^, we characterized P_o_ in the NaOH-EDTA extracts of three aquatic macrophytes and three algae samples by solution ^31^P NMR spectroscopy. Our group also characterized the P forms and C functional groups of the water extracts of the macrophytes collected from the macrophyte-dominated zone in Lake Tai^31^. These studies found the presence of multiple monoester and diester P forms in the aquatic macrophyte and algal biomass, suggesting that P_o_ from the dying and dead biomass of these samples might be transformed into P_i_ that eventually contributes to algal blooming associated with eutrophication.

Three aquatic macrophytes [*Foxtail algae* (A1), *Common reed* (A2), *Verticillata* (A3)] were collected from Lake Tai, and three algal species [*Microcystis* (B1), *Chirorella vulgaris* (B2), *Spirulina* (B3)] were collected from Lake Tai and provided by Institute of Hydrobiology, Chinese Academy of Sciences. Three representative aquatic macrophytes (two submerged macrophytes and one emergent macrophyte) were investigated. Two submerged macrophytes (i. e. A1, A3) and one emergent macrophyte (i. e. A2) were collected in the region of Lake Tai during late October 2010. Whole aquatic macrophytes (i. e. roots, stems, leaves) were collected by use of a plant collector, then washed and took back to laboratory. These field samples were dried to a constant weight at 60 °C, ground, and sieved through a 2-mm screen^21^,^38^. The resulting powder samples were stored at −20 °C until use.

### NaOH-EDTA extractions for solution enzymatic hydrolysis and ^31^P-NMR spectroscopy

Phosphorus extraction was followed the procedure of Feng^13^. Briefly, prepared samples of aquatic macrophytes and algae (0.5 g DM) were extracted with 30 mL of 0.5 M NaOH-25 mM EDTA at 22 °C for 16 h on an end-over-end shaker. After extraction, the slurry solutions were centrifuged at 10000 × *g* for 30 min. Supernatants were filtered through 0.45 μm membrane filters to obtain the NaOH-EDTA extracts. Molybdate-reactive P (i. e. MRP) was measured by the molybdenum blue/ascorbic acid method^40^,^41^. The TP concentration of extracts was determined after digestion with potassium persulfate (K_2_S_2_O_8_) in an autoclave at 121 °C for 30 min^42^. Concentrations of P_o_ in extracts were then calculated as the difference between concentrations of TP and MRP. The remaining extracts were freeze-dried and kept in at −20 °C until ^31^P NMR analysis.

### Enzymatic hydrolysis procedure

Alkaline phosphatase (AP) (EC. 3.1.3.1), phosphodiesterase (PDE) (EC. 3.1.4.1), and crude phytase (Phy) (EC 3.1.3.26) were purchased from Sigma (St Louis, MO). Working solutions of AP and PDE were prepared in Tris-HCl buffer (0.1 M, pH 9.0) at concentrations of 1.0 and 0.02 U mL^−1^, respectively. Crude phytase was purified by dialysis to remove phosphates^2^,^26^. The working phytase solution was prepared in Tris-HCl buffer (0.1 M, pH 7.0) at concentration of 0.06 U mL^−1^. AP was used alone, but PDE was used in combination with AP to achieve complete hydrolysis of diester phosphates. Phytase was used in combination with AP and PDE (0.1 mol·L^−1^, pH 9.0) to ensure that hydrolysis of dissolved P_o_ was as complete as possible^2^.

The enzymatic incubation followed the procedure of Zhu^2^. The incubation solution consisted of 5 mL of pH- adjusted and diluted extracts^2^ and 0.44 mL of the working enzyme solution (AP at pH 9.0, AP + PDE at pH 9.0, or AP + PDE + Phy at pH 7.0) in the appropriate buffer. These mixtures were incubated for 16 h at 37 °C with shaking at 220 rpm. The P_o_ hydrolyzed by each enzyme preparation was calculated as the difference in MRP concentrations determined before and after incubation. Aliquots of the solution was used to determine P_i_ by the molybdenum blue/ascorbic acid method^40^,^41^. Sodium dodecyl sulfate (SDS) was added at 2% (v/v) prior to analysis to prevent enzyme precipitation^41^. A portion of the EH incubation solutions were freeze-dried and subsequently used for ^31^P NMR analysis.

Four classes of P_o_ were quantified based on EH studies of He^21^ and Zhu^2^. They are (1) labile monoester P, which was MRP released by AP; (2) diester P, which was the difference in MRP determined after incubation with AP + PDE, and AP alone; (3) phytate-like P^43^, which was defined as net increase in MRP determined after incubation with AP + PDE + Phy, compared to MRP determined in step (2); and (4) enzyme-stable P that was the part of P_o_ unhydrolyzable by any of the enzyme treatments, it was the difference value between P_o_ with hydrolyzable P_o_ (i. e. labile monoester P, diester P, phytate-like P).

### Solution ^31^P NMR Spectroscopy

Freeze-dried NaOH-EDTA extracts and incubation solutions were dissolved, respectively, in 1 mL 1 M NaOH-0.1 M EDTA/0.1 mL 99% deuterium oxide (D_2_O), and allowed to stand for 30 min at room temperature. Samples were then centrifuged for 30 min at approximately 10000 × *g*, transferred to NMR tubes, and stored at 4 °C before analysis (<24 h). Solution ^31^P NMR spectra were acquired at 161.98 MHz on a Bruker AVANCE 400 MHz spectrometer (Germany) equipped with a 5-mm broadband probe, using a 90° pulse, 0.2102 s acquisition, 5 s pulse delay, and 5-Hz spinning. The number of points was 8192. For each sample, the total NMR experiment lasted 15 to 20 h, collecting 24000 scans. Phosphorus compounds were identified by their chemical shifts, with the orthophosphate peak in all spectra standardized to 6.00 ppm.

### Data Analysis

Data were checked for deviation from normality of variance before analyses. To check whether there was a significant linear relationship between bioavailable P_o_, Pearson correlation coefficients (*r* values, two-tailed) at *p* < 0.01 and *p* < 0.05 were determined using SPSS 11.5. Solution ^31^P NMR Spectra were analyzed by MestReNova software 9.0. Figures were processed using CorelDraw Graphics Suite X4 (Corel Corp.) and OriginPro 8.0 (OriginLab Corp.).

## Additional Information

**How to cite this article**: Feng, W. *et al.* Forms and Lability of Phosphorus in Algae and Aquatic Macrophytes Characterized by Solution ^31^P NMR Coupled with Enzymatic Hydrolysis. *Sci. Rep.* **6**, 37164; doi: 10.1038/srep37164 (2016).

**Publisher’s note**: Springer Nature remains neutral with regard to jurisdictional claims in published maps and institutional affiliations.

## Supplementary Material

Supplementary Information

## Figures and Tables

**Table 1:** P components in the NaOH-EDTA extracts of aquatic macrophytes and algae affected by enzymatic hydrolysis treatments prior to NMR analysis.

Class	P_i_			P_o_															Diester P			C-P	∑*Po*
							Monoester P																			
							Phytate				Other Monoester P															
	Ortho-a	Pyro-	∑*P*_*i*_	chiro-	neo-	other-phy	∑*Phy*	Fru-	α-gly	β-gly	Gua-	Cyc-	Glu-1	Glu-6	_AMP_	_P-acid_	_P-line_	∑*other*	Lipids	DNA	∑*Die*					
A1 + 0e^b^	990(45.9)^c^	52(2.4)	1042(48.3)	424(19.6)	64(3.0)	353(16.3)	841(38.9)	—d	79(3.7)	—	71(3.3)	—	—	—	83(3.8)	—	—	233(10.8)	—	44(2.0)	44(2.0)	—	1117(51.7)
A1 + 1e	2004(92.8)	—	2004(92.8)	—	—	66(3.0)	66(3.0)	—	25(1.2)	—	—	—	—	—	—	6.5(0.3)	—	32(1.5)	58(2.7)	—	58(2.7)	—	155(7.2)
A1 + 2e	1851(85.7)	—	1851(85.7)	—	—	159(7.4)	159(7.4)	—	—	—	—	—	—	—	—	121(5.6)	—	121(5.6)	29(1.3)	—	29(1.3)	—	308(14.3)
A1 + 3e	2068(95.9)	—	2068(95.9)	—	—	—	—	—	—	—	—	—	—	—	—	—	—	—	88(4.1)	—	88(4.1)	—	88(4.1)
A2 + 0e	670(56.6)	10(0.9)	680(57.4)	77(6.5)	68(5.7)	—	145(12.2)	—	96(8.1)	—	—	—	—	—	—	—	190(16.1)	286(24.2)	—	74(6.2)	74(6.2)	—	504(42.6)
A2 + 1e	932(78.7)	—	932(78.7)	—	—	33(2.8)	33(2.8)	—	5(0.4)	—	—	—	—	—	—	5.8(0.5)	2.6(0.2)	13(1.1)	187(15.8)	18(1.5)	206(17.4)	—	252(21.3)
A2 + 2e	1082(91.4)	—	1082(91.4)	—	—	102(8.6)	102(8.6)	—	—	—	—	—	—	—	—	—	—	—	—	—	—	—	102(8.6)
A2 + 3e	1162(98.1)	—	1162(98.1)	—	—	—	—	—	—	—	—	—	—	—	22(1.9)	—	—	22(1.9)	—	—	—	—	22(1.9)
A3 + 0e	832(27.3)	573(18.8)	1405(46.1)	351(11.5)	226(7.4)	—	576(18.9)	881(28.9)	116(3.8)	—	—	—	—	24(0.8)	—	—	—	1021(33.5)	—	49(1.6)	49(1.6)	—	1643(53.9)
A3 + 1e	2902(95.2)	—	2902(95.2)	—	—	—	—	—	—	—	—	—	—	—	—	—	—	—	88(2.9)	58(1.9)	146(4.8)	—	146(4.8)
A3 + 2e	2606(85.5)	—	2606(85.5)	—	—	338(11.1)	338(11.1)	—	—	—	—	—	—	—	—	—	—	—	104(3.4)	—	104(3.4)	—	442(14.5)
A3 + 3e	3048(100)	—	3048(100)	—	—	—	—	—	—	—	—	—	—	—	—	—	—	—	—	—	—	—	—
B1 + 0e	5094(67.5)	137(1.8)	5232(69.3)	596(7.9)	128(1.7)	825(10.9)	1549(20.5)	652(8.6)	—	—	—	—	—	—		—	—	652(8.6)	—	116(1.5)	116(1.5)	—	2318(30.7)
B1 + 1e	5915(78.4)	—	5915(78.4)	14(0.2)	—	270(3.6)	285(3.8)	100(1.3)	47(0.6)	—	—	9(0.1)	—	35(0.5)		—	36(0.5)	227(3.0)	1049(13.9)	73(1.0)	1123(14.9)	—	1634(21.7)
B1 + 2e	6657(88.2)	—	6657(88.2)	—	—	719(9.5)	719(9.5)	—	—	—	—	—	—	—		—	—	—	174(2.3)	—	174(2.3)	—	892(11.8)
B1 + 3e	7183(95.2)	—	7183(95.2)	—	—	—	—	—	—	94(1.2)	—	86(1.1)	—	—		129(1.7)	—	309(4.1)	57(0.8)	—	57(0.8)	—	366(4.9)
B2 + 0e	2646(27.1)	1497(15.3)	4143(42.4)	3498(35.8)	391(4.0)	206(2.1)	4095(41.9)	—	979(10.0)	—	—	431(4.4)	—	—		—	—	1410(14.4)	—	130(1.3)	130(1.3)	—	5636(57.6)
B2 + 1e	7695(78.7)	—	7695(78.7)	—	—	680(7.0)	680(7.0)	—	—	—	—	—	—	—		216(2.2)	19(0.2)	235(2.4)	1119(11.4)	51(0.5)	1170(12.0)	—	2084(21.3)
B2 + 2e	7056(72.2)	—	7056(72.2)	494(5.1)	—	1228(12.6)	1722(17.6)	—	—	—	—	—	—	—		—	—	—	1001(10.2)	—	1001(10.2)	—	2723(27.9)
B2 + 3e	7540(77.1)	—	7540(77.1)	—	—	—	—	—	784(8.0)	—	—	—		—		—	1138(11.6)	1923(19.7)	317(3.2)	—	317(3.2)	—	2239(22.9)
B3 + 0e	4236(32.8)	297(2.3)	4533(35.1)	90(0.7)	594(4.6)	39(0.3)	723(5.6)	2583(20.0)	1227(9.5)	555(4.3)	—	—	1266(9.8)	90(0.7)	—	—	—	5721(44.3)	—	1860(14.4)	1860(14.4)	90(0.7)	8395(65.0)
B3 + 1e	10732(83.1)	220(1.7)	10952(84.8)	—	—	1072(8.3)	1072(8.3)	—	39(0.3)	—	—	—	426(3.3)	—	—	—	—	465(3.6)	220(1.7)	—	220(1.7)	220(1.7)	1963(15.2)
B3 + 2e	10952(84.8)	—	10952(84.8)	116(0.9)	—	1537(11.0)	1537(11.9)	—	—	—	—	—	—	—	—	—	—	—	439(3.4)	—	439(3.4)	—	1976(15.3)
B3 + 3e	12295(95.2)	—	12295(95.2)	129(1.0)	—	—	129(1.0)	—	—	—	245(1.9)	245(1.9)	—	—	—	—	—	491(3.8)	—	—	—	—	620(4.8)

^a^Ortho-: Orthophosphate; Pyro-: Pyrophosphate; ∑*P*: the sum of orthophosphate and pyrophosphate; chiro-: chiro-IHP, neo-: neo-IHP; other-phy: other phytate; ∑*Phy*: the sum of phytate(IHP); Fru-: D-Fructose 6-phosphate; α-gly: α-Glycerophosphate; β-gly: β-Glycerophosphate; Glu-1: Glucose 1-phosphate; Glu-6: D-Glucose 6-phosphate; Gua-: Guanosine 2′ monophosphate; Cyc-: Cytidine 5′ monophosphate; AMP: Adenosine 5′ monophosphate; P-acid: 3-sn phosphatidic acid; P-ine: O-phosphorylethanolamine; ∑*other*: the sum of other monoesters P; Lipids: Lipids phosphate; DNA: deoxyribonucleic acid; ∑*Die*: the sum of diesters P; C-P: Phosphonates; ∑*Po*: the sum of P_o_.

^b^A1, A2, A3, B1, B2, B3: *Foxtail algae, Common reed*, *Verticillata*, *Microcystis, Chirorella vulgaris, Spirulina*; 0e, 1e, 2e, 3e: without enzyme treatments, +AP; +AP + PDE; +AP + PDE + Phy.

^c^Values in parentheses are percentages of individual P compounds in NaOH-EDTA extractable P.

^d^“−”: not detected or negative value.

**Table 2:** Phosphorus forms identified in NaOH-EDTA extracts of aquatic macrophytes and algae by enzymatic hydrolysis.

Code	Sample	P_i_	Total hydrolyzable P_o_	Phytate-like P	labile Monoester P	Diester P	Enzyme-stable P
		% of total P extracted					
A1	*Foxtail algae*	33.4 ± 5.1^a^	26.9 ± 3.4	23.5 ± 2.6	3.4 ± 0.5	—b	39.7 ± 5.4
A2	*Common reed*	58.7 ± 4.8	11.8 ± 2.1	—	8.0 ± 1.5	3.8 ± 0.5	29.5 ± 2.8
A3	*Verticillata*	61.0 ± 3.6	31.2 ± 2.9	18.5 ± 1.4	12.7 ± 0.4	—	7.8 ± 0.1
B1	*Microcystis*	30.0 ± 4.8	41.3 ± 5.8	26.4 ± 5.8	14.9 ± 2.8	—	28.7 ± 4.1
B2	*Chlorella vulgaris*	42.3 ± 5.6	15.5 ± 2.3	12.2 ± 0.9	3.3 ± 0.5	—	42.2 ± 0.8
B3	*Spirulina*	34.2 ± 2.4	42.9 ± 1.8	4.8 ± 0.2	31.3 ± 2.1	6.8 ± 0.5	22.8 ± 1.7
		mg kg ^-1^					
A1	*Foxtail algae*	820 ± 125	660 ± 83	577 ± 64	83 ± 12	—	975 ± 133
A2	*Common reed*	697 ± 57	140 ± 25	—	71 ± 6	45 ± 6	350 ± 33
A3	*Verticillata*	1860 ± 110	951 ± 88	564 ± 43	387 ± 12	—	238 ± 3
B1	*Microcystis*	2429 ± 389	3344 ± 470	2138 ± 470	1206 ± 227	—	2324 ± 332
B2	*Chlorella vulgaris*	3735 ± 494	1369 ± 203	1077 ± 79	291 ± 44	—	3726 ± 71
B3	*Spirulina*	3497 ± 245	4387 ± 184	491 ± 21	3200 ± 215	695 ± 51	2331 ± 174

^a^Mean with standard deviation, *n* = 3.

^b^“−”:not detected or negative value.
