# Supplementary material for: Forms and Lability of Phosphorus in Algae and Aquatic Macrophytes Characterized by Solution 31P NMR Coupled with Enzymatic Hydrolysis
Source: Sci Rep. 2016 Nov 16;6:37164. doi: 10.1038/srep37164 (PMC5111050; doi:10.1038/srep37164)
Supplement: Supplementary Information [file srep37164-s1.pdf]

Supplementary Information

Forms and Lability of Phosphorus in Algae and Aquatic Macrophytes Characterized by Solution ^{31}P NMR Coupled with Enzymatic Hydrolysis

Weiying Feng [a,b], *Yuanrong Zhu* [a*], *Fengchang Wu* [a*], *Zhongqi He* [c], *Chen Zhang* [a],

John P. Giesy[a,d]

Author affiliation:

[a] State Key Laboratory of Environmental Criteria and Risk Assessment, Chinese Research Academy of Environmental Sciences, Beijing 100012, China;

[b] College of Water Sciences, Beijing Normal University, Beijing 100875, China;

[c] USDA-ARS, Southern Regional Research Center, New Orleans, LA 70124, USA

[d] Department of Biomedical Veterinary Sciences and Toxicology Centre, University of Saskatchewan, Saskatoon, SK S7N 5B3, Canada

Corresponding author:

[*]To whom correspondence may be addressed

Prof. Fengchang Wu and Dr. Yuanrong Zhu

Telephone number: +86-10-84915312

Fax number: +86-10-84931804

E-mail: wufengchang@vip.skleg.cn;

zhuyuanrong07@mails.ucas.ac.cn

Supplementary Information

S.I. Table 1 Chemical shifts of monoester P peaks indentified in NaOH-EDTA extracts of aquatic macrophytes and algae

Class	Chemical shift(ppm)	Class	Chemical shift(ppm)
chiro-IHP[a], 2e/4a[b]	6.55,5.28,3.96	α-D-Glucose 1-phosphate	3.336
chiro-IHP, 4e/2a[b]	6.21,4.75,4.33	D-Glucose 6-phosphate	5.246
neo-IHP,4e/2a	6.40,4.26[c]	Guanosine 2’	4.500
myo-IHP, 2e/4a	5.491,4.522,4.127,4.020	Cytidine 5’	4.397
D-Fructose 6-phosphate	4.617	3-sn phosphatidic acid	4.866
α-Glycerophosphate	4.880	O-phosphorylethanolamine	4.580
β-Glycerophosphate	4.529	Adenosine 5’	4.405

[a] IHP, inositol hexakisphosphate.

[b] 2e/4a,2 equatorial/4 axial conformation; 4e/2a, 4 equatorial/2 axial conformation.
